# Integrative analysis of long extracellular RNAs reveals a detection panel of noncoding RNAs for liver cancer

**DOI:** 10.7150/thno.48206

**Published:** 2021-01-01

**Authors:** Yumin Zhu, Siqi Wang, Xiaochen Xi, Minfeng Zhang, Xiaofan Liu, Weina Tang, Peng Cai, Shaozhen Xing, Pengfei Bao, Yunfan Jin, Weihao Zhao, Yinghui Chen, Huanan Zhao, Xiaodong Jia, Shanshan Lu, Yinying Lu, Lei Chen, Jianhua Yin, Zhi John Lu

**Affiliations:** 1 MOE Key Laboratory of Bioinformatics, Center for Synthetic and Systems Biology, School of Life Sciences, Tsinghua University, Beijing 100084, China.; 2 Department of Maternal, Child and Adolescent Health, School of Public Health, Anhui Medical University, MOE Key Laboratory of Population Health Across Life Cycle, NHC Key Laboratory of Study on Abnormal Gametes and Reproductive Tract, Anhui Provincial Key Laboratory of Population Health and Aristogenics, No 81 Meishan Road, Hefei 230032, Anhui, China.; 3 Department of Surgery, Eastern Hepatobiliary Surgery Hospital, Navy Medical University, Shanghai 200433, China.; 4 Department of Epidemiology, Faculty of Navy Medicine, Navy Medical University, Shanghai 200433, China.; 5 International Co-operation Laboratory on Signal Transduction, Eastern Hepatobiliary Surgery Institute, Second Military medical University, Shanghai 200438, China.; 6 National Center for Liver Cancer, Shanghai 201805, China.

**Keywords:** circular RNA, extracellular RNA, liquid biopsy, noncoding RNA, RNA biomarker, cancer

## Abstract

**Rationale:** Long extracellular RNAs (exRNAs) in plasma can be profiled by new sequencing technologies, even with low abundance. However, cancer-related exRNAs and their variations remain understudied.

**Methods:** We investigated different variations (i.e. differential expression, alternative splicing, alternative polyadenylation, and differential editing) in diverse long exRNA species (e.g. long noncoding RNAs and circular RNAs) using 79 plasma exosomal RNA-seq (exoRNA-seq) datasets of multiple cancer types. We then integrated 53 exoRNA-seq datasets and 65 self-profiled cell-free RNA-seq (cfRNA-seq) datasets to identify recurrent variations in liver cancer patients. We further combined TCGA tissue RNA-seq datasets and validated biomarker candidates by RT-qPCR in an individual cohort of more than 100 plasma samples. Finally, we used machine learning models to identify a signature of 3 noncoding RNAs for the detection of liver cancer.

**Results:** We found that different types of RNA variations identified from exoRNA-seq data were enriched in pathways related to tumorigenesis and metastasis, immune, and metabolism, suggesting that cancer signals can be detected from long exRNAs. Subsequently, we identified more than 100 recurrent variations in plasma from liver cancer patients by integrating exoRNA-seq and cfRNA-seq datasets. From these datasets, 5 significantly up-regulated long exRNAs were confirmed by TCGA data and validated by RT-qPCR in an independent cohort. When using machine learning models to combine two of these validated circular and structured RNAs (*SNORD3B-1, circ-0080695*) with a miRNA (*miR-122*) as a panel to classify liver cancer patients from healthy donors, the average AUROC of the cross-validation was 89.4%. The selected 3-RNA panel successfully detected 79.2% AFP-negative samples and 77.1% early-stage liver cancer samples in the testing and validation sets.

**Conclusions:** Our study revealed that different types of RNA variations related to cancer can be detected in plasma and identified a 3-RNA detection panel for liver cancer, especially for AFP-negative and early-stage patients.

## Introduction

Recent studies have shown that circulating cell-free RNAs (cfRNAs) in liquid biopsies are promising biomarkers 1-3. RNA markers have several advantages, including sensitivity, tissue specificity, and low cost for detection 4. cfRNAs are also called extracellular RNAs (exRNAs) and include RNAs located in microvesicles (MV), exosomes, and non-vesicular ribonucleic acid protein complexes (RNPs) 5. Because of the protection of the exosome membrane, binding proteins or self-structure, certain exRNAs are able to resist degradation by RNases and remain stable in body fluid.

Previous exRNA biomarker studies have primarily focused on miRNAs 6. Many extracellular miRNAs have been identified as predictive biomarkers for various cancers 7, 8. In addition to miRNAs, many other species of RNAs have also been detected among exRNAs, including transfer RNA (tRNA), Y RNA, circular RNA (circRNA), and fragments of messenger RNA (mRNA) and long noncoding RNA (lncRNA) 12, 14. As many miRNAs are enriched in exosomes, other RNAs, including tRNA and Y-RNA fragments, have largely been found in RNPs 13. The clinical value of various types of exRNAs has been recognized 15. For example, *PD-L1*, an mRNA transcript associated with the response to treatment for non-small lung cancer showed reliable clinical performance in plasma 16. A lncRNA, *PCA3*, was identified as a prostate cancer biomarker in urine 18. Several other lncRNAs in plasma were identified as HCC biomarker candidates 19. The expression level of *RN7SL1*, a signal recognition particle RNA (srp RNA), was found to be correlated with breast cancer progression 20 and HCC diagnosis and prognosis 19. A circRNA, *circMYBL2*, regulates *FLT3* translation by recruiting PTBP1 to promote FLT3-ITD AML progression 24. A 3-circRNA signature was identified as a noninvasive biomarker for the diagnosis of colorectal cancer 25.

In addition to change in abundance of RNAs, post-transcriptional RNA isoform variations are also associated with cancer development and progression, and such variations could serve as cancer biomarkers 1. For instance, several studies found that differential RNA editing events between cancer patients and healthy people may serve as biomarkers or therapeutic targets for cancer 26, 27. Shen et al. suggested that alternative splicing was correlated with patient survival time 28. Xia et al. identified many genes implicated in cancer pathogenesis by studying their alternative polyadenylation (APA) 29. These events were initially identified from studies of tumor tissues; however, recent studies have assessed small RNA editing and mRNA splicing events in plasma, urine, and other body fluids as biomarkers for the diagnosis of cancers and other diseases 30, 31.

In comparison with small RNA-seq, long RNA-seq can provide more information than abundant level. For instance, long RNA-seq can be used to detect various types of RNAs, including circRNA and lncRNA, as well as post-transcriptional variations like alternative splicing and editing of mRNAs. Therefore, in this study, we investigated the expression profiles of long exRNAs in multiple cancer types in order to find cancer-related long exRNA species and their variations in plasma samples. Moreover, we validated selected liver cancer variations with multiple cohorts and various methods, including exosomal RNA-seq (exoRNA-seq), TCGA tissue RNA-seq, cell-free RNA-seq (cfRNA-seq), exosome enrichment assays, and RT-qPCR. Our findings revealed a multi-RNA panel that could be used for the diagnosis of liver cancer, especially for alpha feto-protein (AFP)-negative and early stage patients.

## Results

### Different RNA variations can be detected in long exoRNA-seq data from multiple cancer types

**Figure 1:**
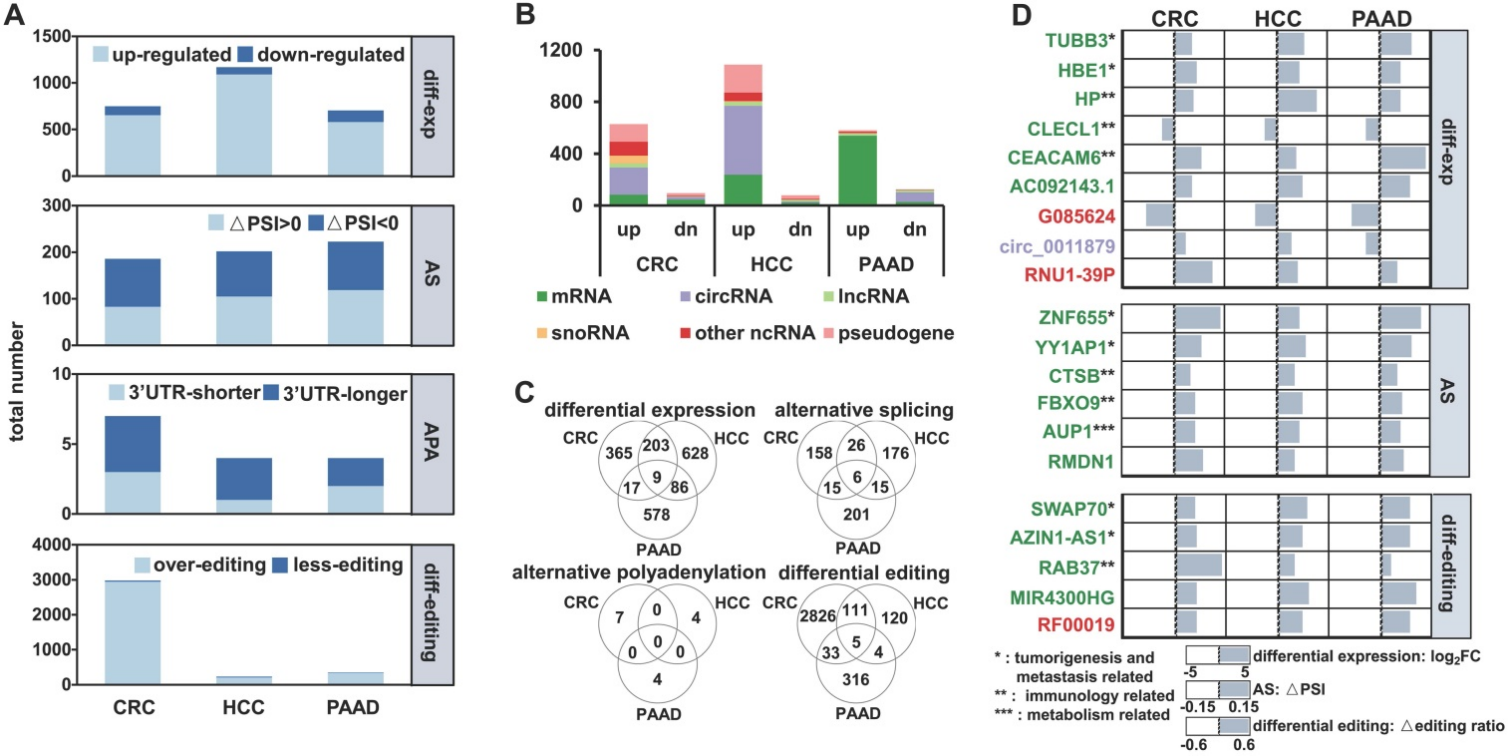
** Multiple variations of long exosomal RNAs in different cancer types. (A)** Multiple variations of long exRNAs identified in three cancer types (CRC: colorectal cancer, HCC: hepatocellular carcinoma, PAAD: pancreatic adenocarcinoma) using healthy donors as control. The dark blue and light blue indicate the opposite pattern of RNA variations, including differential expression, alternative splicing (AS), alternative polyadenylation (APA), and differential editing. FC: fold-change, FDR: false discovery rate, PSI: percent spliced in index, UTR: untranslated region. **(B)** Numbers of differentially expressed RNAs for different RNA types. up: up-regulation, dn: down-regulation. **(C)** Overlap of differential expression, alternative splicing, alternative polyadenylation, differential editing events among three types of cancer. **(D)** List of common differential expression, alternative splicing, and differential editing events among three cancer types.

We first analyzed exosomal RNA-seq (exoRNA-seq) data from patients with three types of cancer and healthy donors (HDs) in 79 datasets that were curated by exoRBase 32, including 12 colorectal cancer (CRC) patients, 21 hepatocellular carcinoma (HCC) patients, 14 pancreatic adenocarcinoma (PAAD) patients, and 32 HDs. In total, using the HDs as control, we identified differential expression, alternative splicing (AS), APA, and differential editing events in CRC, HCC, and PAAD patients (Figure 1A). For instance, 749, 1,168 and 703 differentially expressed genes (|log2(fold-change)| ≥ 1 and false discovery rate (FDR) value ≤ 0.05) were identified in CRC, HCC, and PAAD patients, respectively. We found that a large proportion of differentially expressed genes were circRNAs and pseudogenes (Figure 1B), which was in accordance with previous studies 15, 33.

There were only a few common events (nine differential expression, six AS, and five differential editing events) among the RNA variations identified in CRC, HCC, and PAAD patients (Figure 1C), suggesting that these regulatory events have a high degree of specificity for each cancer type. The genes identified in all three groups of patients are involved in tumorigenesis and metastasis, immune, and metabolism-related pathways, which are closely linked to the occurrence and development of cancer (Figure 1D). Furthermore, 19 of the 20 variations had consistent patterns (Log_2_FC > 0 or < 0, △PSI > 0 or < 0 and △editing ratio > 0 or < 0) among the CRC, HCC, and PAAD patients (Figure 1D).

### The cancer specific exRNA variations are enriched with cancer genes and functionally coherent

**Figure 2:**
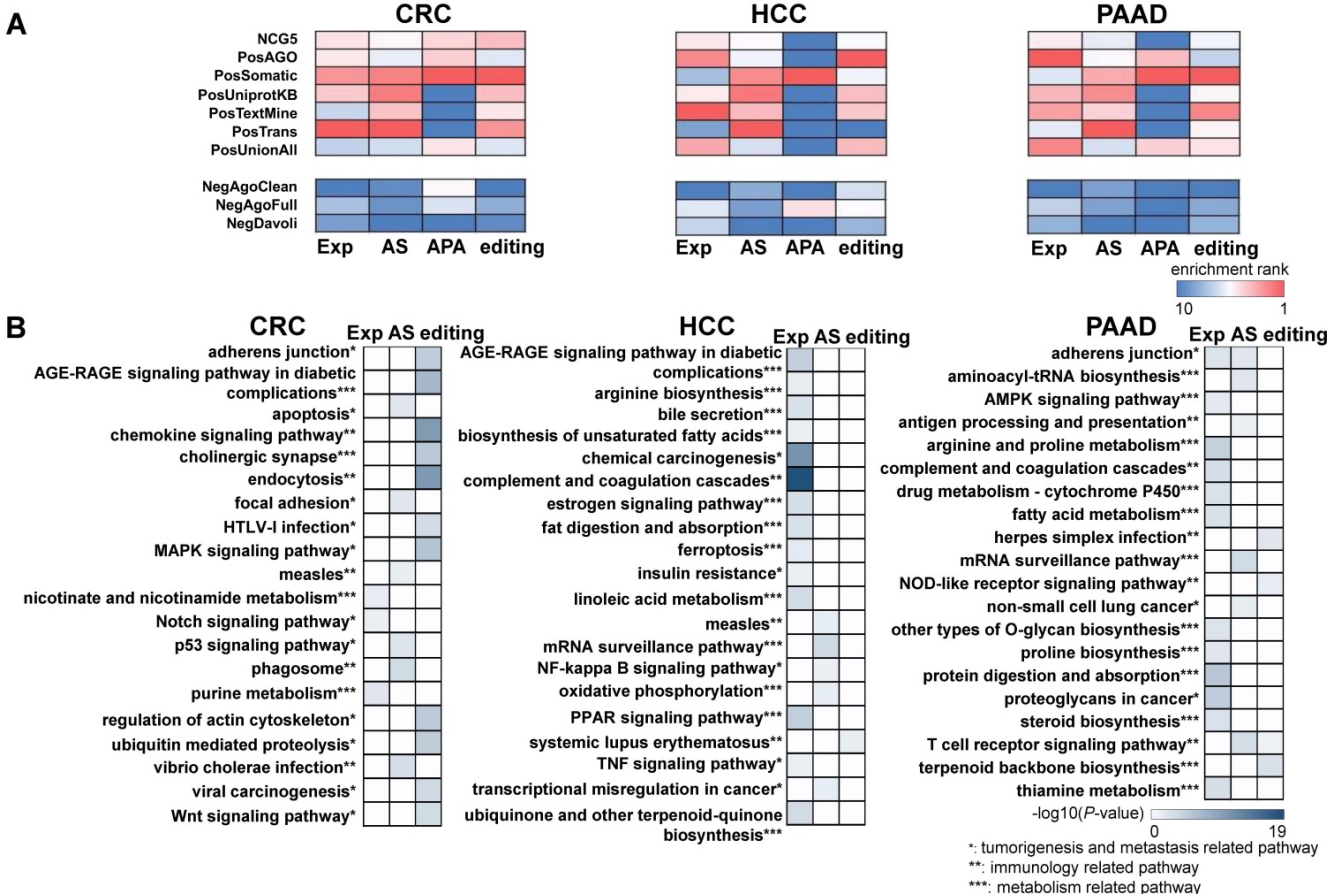
** The exosomal RNA variations are enriched in cancer genes and cancer related pathways. (A)** Cancer gene enrichment (top heatmaps) and non-cancer gene enrichment (bottom heatmaps) of RNA variations for three cancer types. NCG5, PosAGO, PosSomatic, PosUniprotKB, PosTextMine, PosTrans, PosUnionAll are the cancer gene lists, while NegAgoClean, NegAgoFull, and NegDavoli are the non-cancer gene lists (see Methods). **(B)** Top 20 enriched KEGG pathways of differential expression, alternative splicing, and differential editing events for three cancer types.

In addition to the variations found in CRC, HCC, and PAAD patients, we also evaluated the functional relevance of variations specific to each cancer type. We curated known cancer genes (positive controls), as well as non-cancer genes (negative controls) 34, and then calculated the enrichment of extracellular RNA (exRNA) variations among them (see Methods). We overlapped each type of exRNA variation and the cancer gene lists, and the enrichment was calculated as the fraction of overlapped gene members in each cancer gene set. From a general view, four types of exRNA variations were consistently enriched across the seven positive gene lists in comparison with the negative control lists for each of the three types of cancer (Figure 2A). The enrichment pattern of the APA events in HCC and PAAD patients was unclear, because the number of identified APA events was insufficient for enrichment analyses.

We further investigated functional coherence by calculating enrichment in curated pathways from the Kyoto Encyclopedia of Genes and Genomes (KEGG) 35. APA genes were excluded because the number of these genes was insufficient to allow KEGG analysis. The top 20 enriched pathways were tumorigenesis and metastasis-related, immune-related, and metabolism-related pathways across all three cancer types (Figure 2B). The set of enriched pathways showed similarity among CRC, HCC, and PAAD, indicating shared mechanisms among malignancies. In addition, different enriched pathways were identified for each type of RNA variation, indicating complementarity between different regulatory events. The enrichment profiles described above suggest that some of the identified RNA variations could serve as cancer biomarkers in plasma.

### Recurrent exRNA variations were identified as potential biomarkers for liver cancer

**Figure 3:**
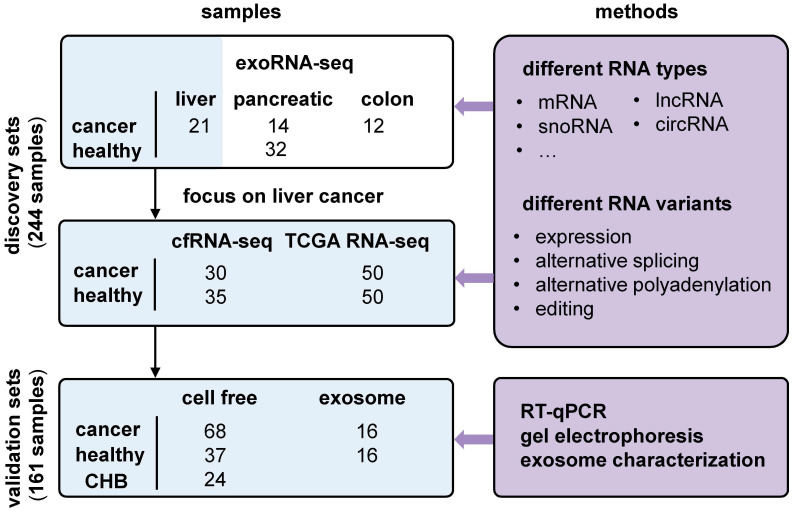
** Overview of experimental design and integrative analysis.** Three discovery sets, exosomal RNA-seq (exoRNA-seq) data from exoRBase, self-profiled cell free RNA-seq (cfRNA-seq) data and tissue RNA-seq data from TCGA, were used to discover candidate biomarkers. Two validation sets (qRT-PCR data of cell free and exosomal RNAs) were used for experimental verification. Different RNA types and variations were assayed. Several experimental methods were applied to validate the candidate biomarkers. CHB: chronic hepatitis B.

We used liver cancer as an application example and identified recurrent variations in multiple datasets (Figure 3). First, we profiled cell-free RNA-seq (cfRNA-seq) data to identify recurrent RNA variations in both exosome/Extracellular Vesicles (EV) and cell-free environments. Meanwhile, we curated clinical information (i.e. stages of cancer) for the cfRNA-seq samples, because the exoRNA-seq datasets did not include clinical information like the cancer stage of the patients. Next, we used TCGA tissue data to confirm the expression patterns of recurrent RNA variations in tumors. The exoRNA-seq, cfRNA-seq and TCGA datasets were designated as discovery datasets. Finally, we used RT-qPCR, gel electrophoresis and exosome characterization to validate the selected biomarker candidates in independent cohorts to generate independent cohort validation datasets.

**Figure 4:**
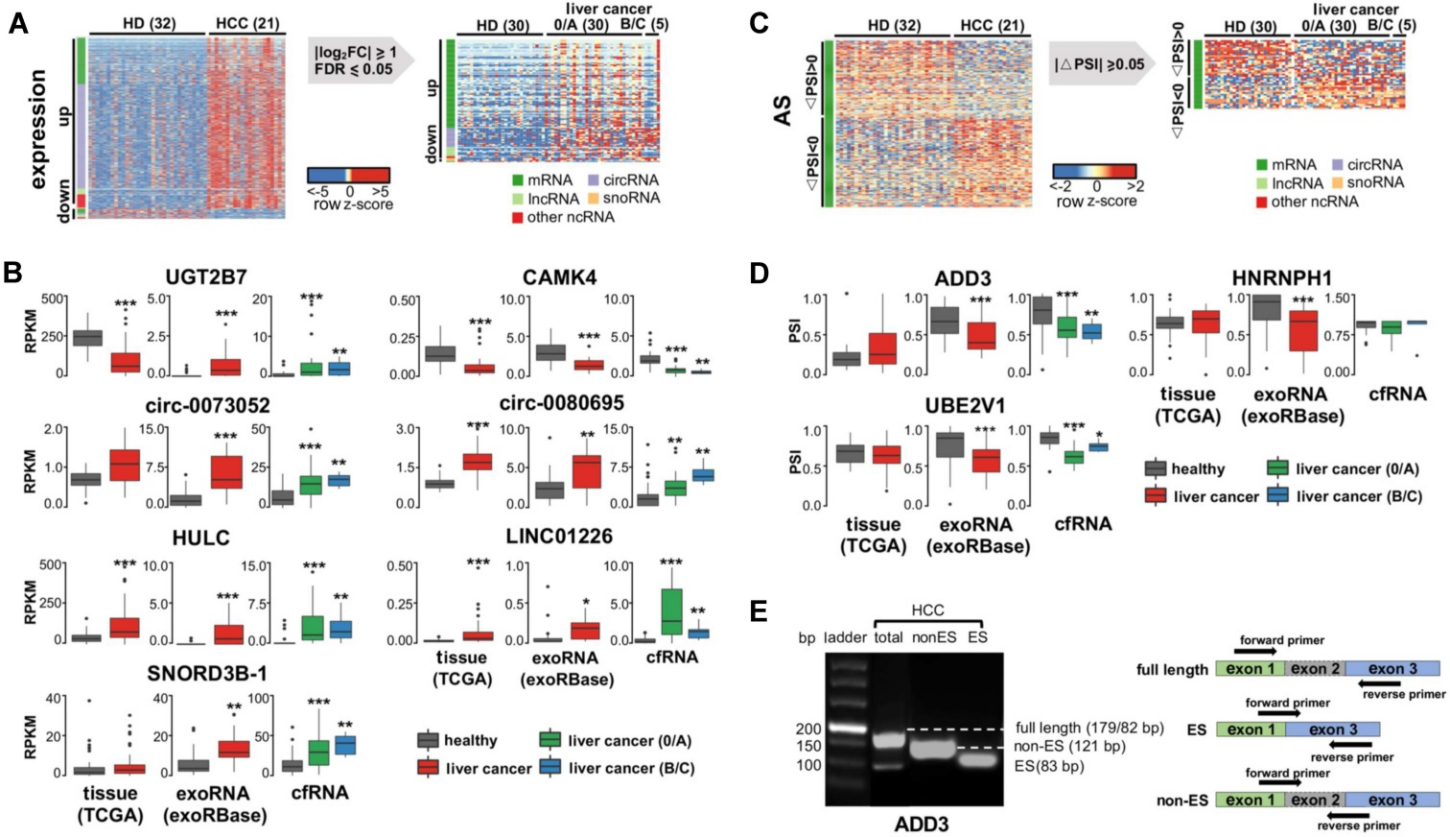
** Identify recurrent exRNA variations from multiple datasets for liver cancer. (A)** Differentially expressed exRNAs of liver cancer idenfied from both exoRNA-seq and cfRNA-seq data, using healthy donor as control. **(B)** Expression level of seven differentially expressed exRNAs in tissue RNA-seq data (TCGA), exoRNA-seq data (exoRBase), and cfRNA-seq data. ***: P-value < 0.001, **: P-value < 0.01, *: P-value < 0.05, Wilcoxon rank sum test. **(C)** Alternatively spliced exRNAs idenfied from both exoRNA-seq and cfRNA-seq data. **(D)** Inclusion level of three alternatively spliced exRNAs in tissue RNA-seq data (TCGA), exoRNA-seq data (exoRBase), and cfRNA-seq data. ***: P-value < 0.001, **: P-value < 0.01, *: P-value < 0.05, Wilcoxon rank sum test. **(E)** Representative gel electrophoresis image showing alternative splicing of ADD3 (left); primers designed for validating alternative splicing (right). Three kinds of primers were designed for full length, exon skipping (ES), and non-exon skipping/exon inclusion (Non-ES).

We previously identified 1,168 differentially expressed genes in exoRNA-seq data (Figure 1A). We excluded pseudogenes from subsequent assays because they had ambiguity with their parent genes, leaving a set of 926 differentially expressed genes. Next, we profiled cfRNAs in 65 plasma samples (Table S1) to identify recurrent RNAs dysregulated in both cell-free and exosome/EV environments. The read distribution of the cfRNA-seq data was similar to that of the exoRNA-seq data (Figure S2). Among the differentially expressed genes identified in the exoRNA-seq data, 122 genes were also differentially expressed in the cfRNA-seq data (Figure 4A).

Considering fold-change, FDR, and biological function (see Methods), we selected 7 of 122 recurrently differentially expressed genes for further assays, including 2 mRNAs (*UGT2B7* and *CAMK4*), 2 circRNAs (*circ-0073052* and *circ-0080695*), 2 lncRNAs (*HULC* and *LINC01226*), and 1 snoRNA (*SNORD3B-1*). Additionally, we collected 50 tumor and paired normal tissue RNA-seq datasets of liver cancer from the TCGA database and evaluated the expression levels of these genes in tissues. The results of these experiments revealed that 6 of the 7 selected genes had similar expression patterns in the TCGA data and the tissue samples (Figure 4B).

As we did for differential expression, we assayed recurrent variations for the presence of AS, APA, and differential RNA editing. We found no recurrence of differential APA or RNA editing events (Figure S4). For AS events, we selected 3 events (in *ADD3*, *HNPNPH1* and *UBE3V1*) (see Methods in detail) for further validation. However, none of these 3 genes had the same AS pattern in multiple samples from the TCGA or exoRNA-seq data (Figure 4D), although the AS events were clearly detected in some individuals (Figure 4E). Most of these variations were detected in mRNAs. These results suggest that mRNA fragments and their variations may not be stably detected in individual plasma samples.

**Figure 5:**
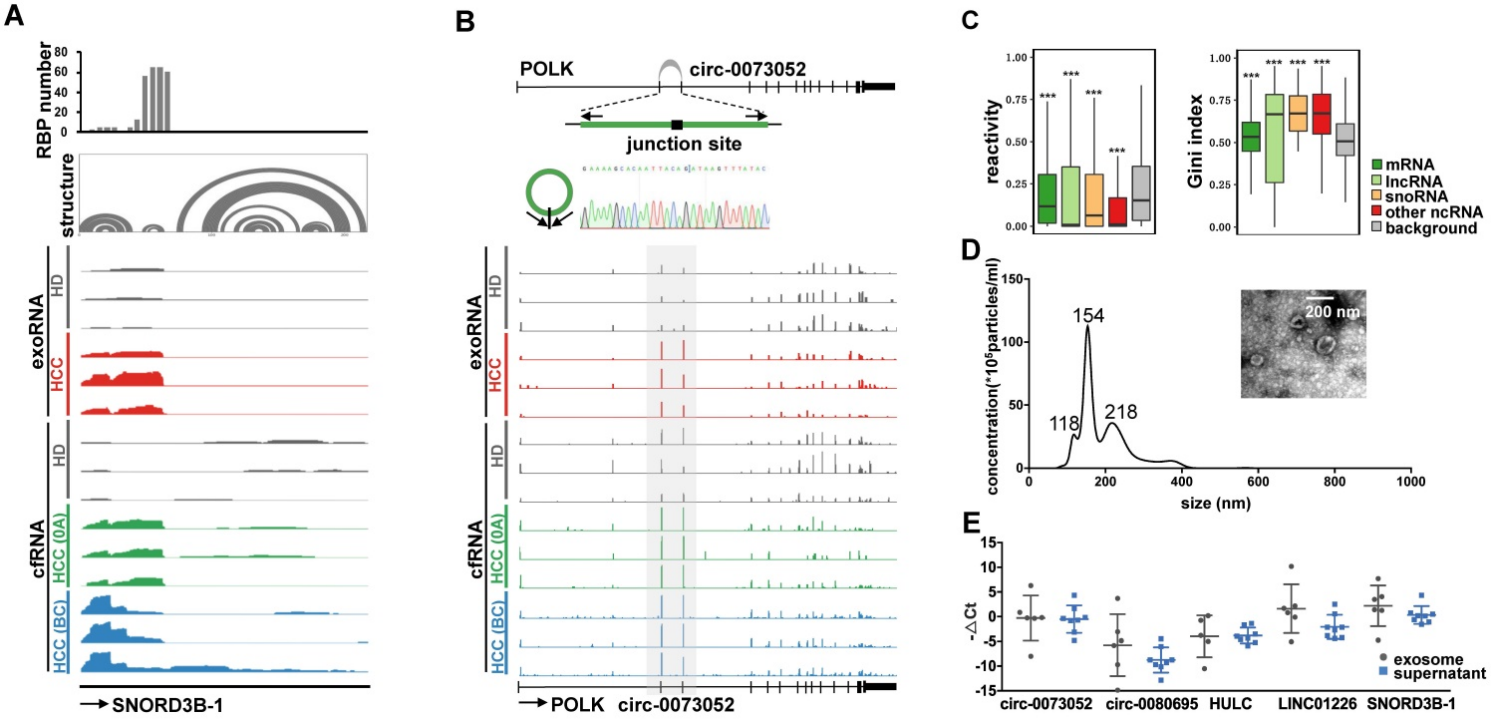
** Examples of the selected exRNA biomarkers for liver cancer detection. (A)** RBP binding profile, RNA secondary structure and reads distribution of exoRNA-seq and cfRNA-seq for SNORD3B-1. **(B)** The genomic locus of *circ-0073052* in *POLK* gene. The supported unique reads are presented. The expression of *circ-0073052* was validated by RT-qPCR followed by sanger sequencing. Arrows represent divergent primers binding to the genome region of *circ-0073052*. Reads distributions of *POLK* and *circ-0073052* for exoRNA-seq and cfRNA-seq show the differential expression pattern (in the gray box) of *circ-0073052* instead of *POLK*. **(C)** Enrichment of RNA secondary structure in up-regulated mRNAs, lncRNAs, snoRNAs, and other ncRNAs. Comparison of icSHAPE reactivities (left box-plot) and gini indexes (right box-plot) between up-regulated RNAs identified by exoRNA-seq and shuffled background RNAs. Higher icSHAPE reactivity represents more unpaired bases in a RNA. Higher gini index represents that RNA is more structured. ***: P-value < 0.001, **: P-value < 0.01, *: P-value < 0.05, Wilcoxon rank sum test. **(D)** Characterizations of exosomes purified from plasma mixtures. The curve indicates the diameter distribution of exosomes by Nanosight. The transmission electron micrograph shows the external morphology of exosomes. **(E)** Relative expression levels measured by RT-qPCR of the 5 selected long exRNAs in exosome and supernatant isolated from the same samples. No significant difference between exosome and supernatant (Wilcoxon rank sum test).

Finally, we validated 10 candidate events (7 differential expression genes and 3 AS events) using RT-qPCR in the 26 samples that were previously sequenced (Figure S4). Actually, 8 (5 differential expression genes and 3 AS events) out of the 10 candidate events were consistent between the results of RT-qPCR and sequencing. We also found that the noncoding RNA candidates were more consistent than the mRNA candidates in terms of both differential expression and AS. In other words, the candidate noncoding RNAs could serve as more stable biomarkers in comparison with the candidate mRNAs, perhaps due to their self-structure or protein protection. Thus, although we were able to develop a combinatory model using all 10 candidates together (Figure 5), we selected only the 5 noncoding RNAs (*circ-0073052*, *circ-0080695*, *SNORD3B-1, LINC01226* and *HULC*) as potential exRNA biomarkers for further analysis and validation.

### The selected exRNAs include various circular and structured noncoding RNAs

The 5 selected exRNAs include various types of RNAs, including lncRNA, circRNA, and snoRNA. Among them, the lncRNA *HULC* has already been established as a diagnostic biomarker of HCC 36. SnoRNAs also show clinical significance and play important roles in HCC 40. Collectively, we selected five genes as potential exRNA biomarkers for further analysis and validation, including known biomarkers *HULC* and novel biomarker candidates *LINC01226*, *circ-0073052*, *circ-0080695*, and *SNORD3B-1*.

We used two types of exRNAs as examples to demonstrate the potential mechanisms by which these RNAs might be protected from degradation. For *SNORD3B-1*, we collected the structure profile from the Rfam database and RBP binding hotspots from the POSTAR2 database 44, 45. Consistent with the read distribution patterns of the exosome exoRNA and plasma cfRNA sequencing data, the 5′ region of *SNORD3B-1* was enriched with RBP binding sites and RNA secondary structures (Figure 5A). With regard to *circ-0073052*, unlike linear RNAs, its covalently closed cyclic structure might be responsible for its stability in plasma. The genomic structure of *circ-0073052* contains the second and third exon of the *POLK* gene. A distinct product of the expected size was amplified using outward-facing primers and confirmed by Sanger sequencing (Figure 5B). In agreement with the RNA-seq results, *circ-0073052* was significantly more abundant than *POLK* in liver cancer patients, while there was no significant difference in healthy exosomes and plasma samples. The abundance of *circ-0073052* in liver cancer patients was higher than that of HDs. The expression level of the linear *POLK* RNA transcript showed no significant differences between HDs and HCC patients, demonstrating that up-regulation of *circ-0073052* was not influenced by its host gene. These results suggest that *SNORD3B-1* and *circ-0073052* are reliable HCC plasma biomarkers, and RNAs with high abundance might be stabilized in plasma through the formation of stable RNA secondary structures or association with RNA binding proteins.

To explore whether the up-regulated RNAs in plasma were stabilized by RNA secondary structure, we calculated the structural reactivity and Gini index of each nucleotide using *in vivo* icSHAPE data from the HEK293 cell line 46. Background RNAs were generated by shuffling RNAs among all detected transcripts. We found that up-regulated RNAs in plasma were significantly more structured than background RNAs for mRNAs, lncRNAs, snoRNAs and other noncoding RNAs (Figure 5C).

### Extracellular location of the selected exRNAs

We isolated exosomes from plasma to determine the extracellular location of the 5 selected exRNAs (see Methods). The shape and size of exosomes were typical of extracellular vesicles (EVs) (Figure 5D). The exosomes and supernatant were isolated from the same samples, and we found no significant differences in the abundance of these 5 exRNAs between exosomes and the plasma supernatant by Wilcoxon rank sum test (Figure 5E). This result indicates exosomes do not need to be isolated prior to evaluating the abundance of these exRNAs. These findings also show that the candidate biomarkers can be conveniently measured without purifying exosomes, which is an important consideration for liquid biopsy biomarkers. Furthermore, these results suggest that protection by the exosome membrane may not be the main reason that the five selected noncoding RNAs escaped RNA degradation, because certain RNAs can avoid RNase degradation via RNA binding proteins or RNA secondary structures 47.

### Independent validation of the selected exRNA biomarkers for liver cancer

**Figure 6:**
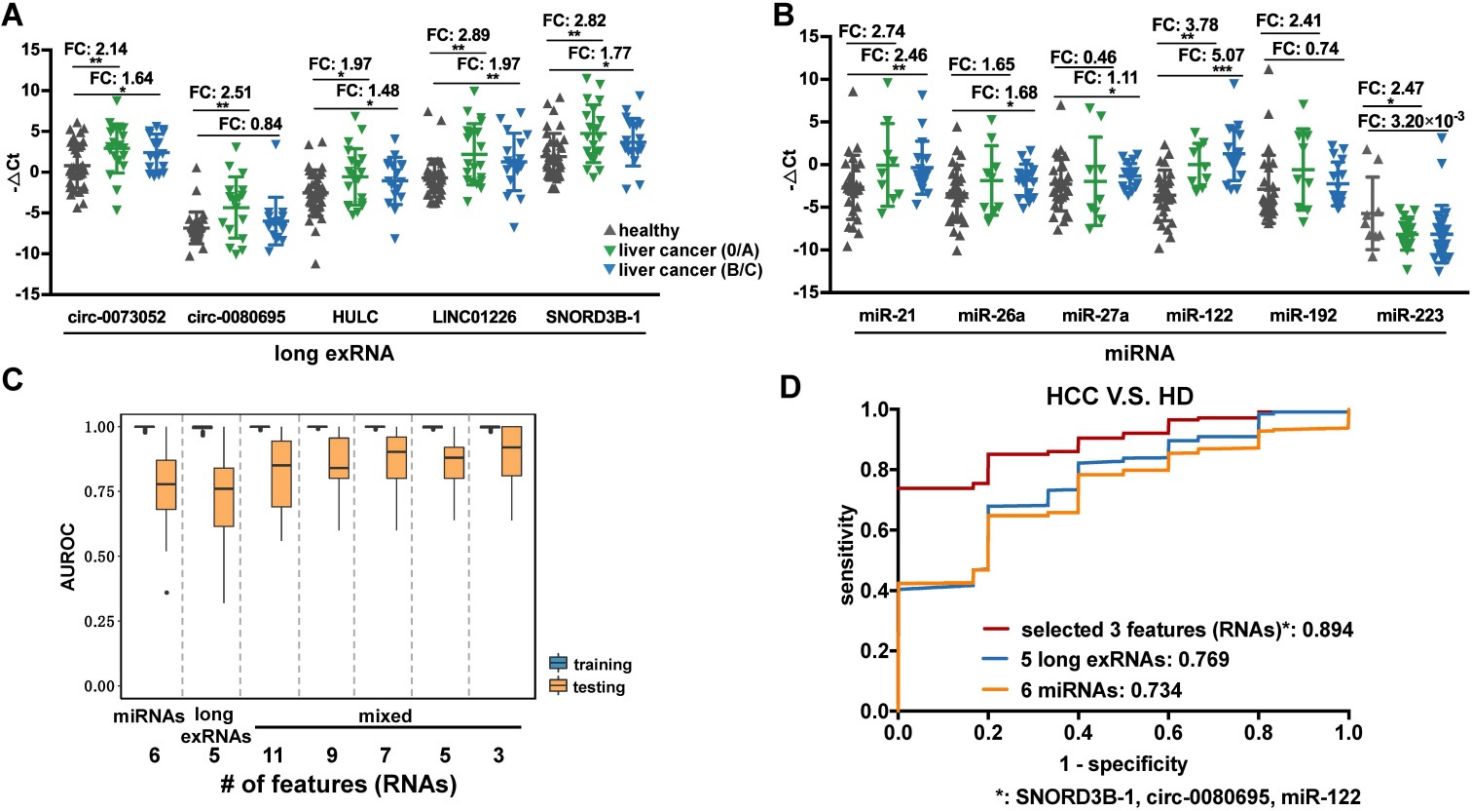
** Performance of the selected long exRNAs and known miRNAs on liver cancer detection. (A)** Validation of 5 selected long exRNAs by RT-qPCR in an independent cohort, plasma samples of 38 HCC patients V.S. 37 Healthy Donors (HDs). ***: P-value < 0.001, **: P-value < 0.01, *: P-value < 0.05, Wilcoxon rank sum test. **(B)** Validation of 6 previously published miRNA biomarkers by RT-qPCR in a subset of the cohort (26 of 38 HCCs; 26 of 37 HDs). ***: P-value < 0.001, **: P-value < 0.01, *: P-value < 0.05, Wilcoxon rank sum test. **(C)** AUROC values of 5 long exRNAs; 6 miRNAs; and 11, 9, 7, 5, 3 out of all RNAs when classifying HCCs from HDs with Random Forest models. We used 5-fold cross-validation and repeated it 10 times by re-shuffling the data. **(D)** Average ROC curves of the selected 3 RNAs, 5 long exRNAs, and 6 miRNAs. The AUC values are also labeled under the curves.

Five exRNAs were selected as potential liver cancer biomarkers based on evidence from the analyses described above. We further validated and compared them with known miRNA biomarkers 11, 48 using RT-qPCR in an independent cohort including 75 plasma samples (38 HCCs V.S. 37 HDs, Table S1). Here, the 5 selected exRNAs were labeled as long exRNAs and compared to miRNAs (Figure 6A). The 5 long exRNAs were all significantly up-regulated in early-stage HCC patients, and 4 of 5 long exRNAs showed significant changes in late-stage HCC patients (Figure 6A), suggesting that these 5 long exRNAs are reliable biomarkers for liver cancer diagnosis.

Previous studies identified miRNAs as HCC diagnosis biomarkers, so 6 known miRNA biomarkers 11, 48 of liver cancer were validated in 52 of the 75 samples used in the analysis of long exRNAs described above. The expression patterns of the 6 selected miRNA biomarkers were consistent with previous reports (5 of 6 miRNAs showed significant up-regulation in HCC patients in comparison with HDs), which confirmed the effectiveness of these biomarkers. However, only 2 (*miR-122* and *miR-223*) of the 6 miRNAs 11, 48 were significantly up-regulated in early-stage HCC patients in comparison with HDs (Figure 6B). In summary, the HCC detection performance of the 5 selected long exRNAs was better than that of 6 established miRNA biomarkers 11, 48, especially for early-stage HCC detection, when HDs were used as controls.

### A 3-RNA panel for the diagnosis of liver cancer, especially AFP-negative patients

**Figure 7:**
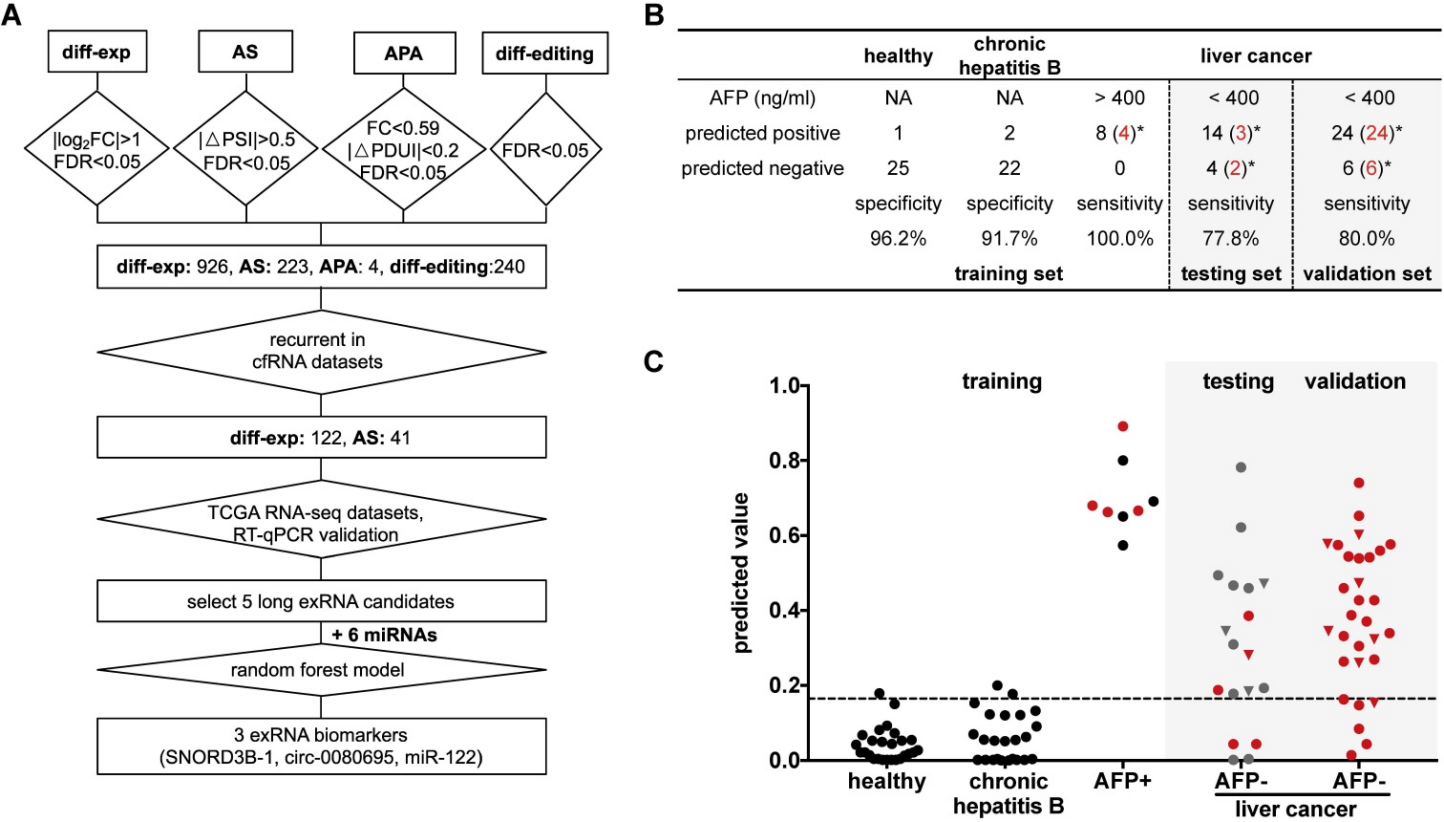
** Detection panel of 3 noncoding RNAs for the AFP-negative and early-stage liver cancer. (A)** Workflow chart of identifying a 3-RNA panel for detecting liver cancer in plasma. Rectangular box indicates the type and quantity of RNA variation. Diamond indicates the screening method and cut-off. **(B)** Performance of the 3-RNA panel (SNORD3B-1, *circ-0080695*, *miR-122*) in training, testing and validation sets (model: Random Forest). Trained on alpha feto-protein (AFP) positive (AFP > 400 ng/ml) patients (HCCs), Chronic hepatitis B patients (CHBs) and healthy donors (HDs); tested and validated on AFP negative (AFP < 400 ng/ml) patients (HCCs). *: early stages (0/A) are labeled in red. NA: Not available. **(C)** Predicted values of the 3-RNA panel (model: Random Forest). The cutoff of the predicted value is defined by requiring > 95% specifity of healthy donors in the training set. Triangle points represent patients with 20 ng/ml < AFP ≤ 400 ng/ml. Red points represent patients of early stages (0/A).

We further optimized a subset of the 5 long exRNAs and 6 miRNAs 11, 48 described above as a diagnostic panel for liver cancer, particularly for alpha feto-protein (AFP)-negative patients. First, we divided the RT-qPCR data described above (52 samples: 26 HCCs V.S. 26 HDs) into 50 class-balanced training and testing sets (see Methods). The area under the receiver operating characteristic curve (AUROC) distribution of random forest models was measured by a 5-fold cross-validation with 10 repetitions. For the different feature combinations of the 11 selected RNAs, we determined the AUROC distributions of 3, 5, 7 and 9 RNAs using 50 testing sets. A 3-RNA panel (*SNORD3B-1, circ-0080695,* and* miR-122*) obtained the highest average AUROC (89.4%) in comparison with a set of 5 long exRNAs (76.9%), a set of 6 miRNAs (73.4%) 11, 48 and other sets of feature combinations (Figure 6C-D). Therefore, we selected the combination of *SNORD3B-1, circ-0080695,* and* miR-*122 as a 3-RNA detection panel for liver cancer (Figure 7A).

Next, we evaluated the performance of the 3-RNA panel for the diagnosis of AFP-negative patients. Serum AFP has been widely used as a non-invasive biomarker for the diagnosis of liver cancer, but it has been demonstrated to show poor sensitivity 49. Here, we observed that only a small proportion of patients was AFP-positive (>400 ng/mL). In order to evaluate the performance of the 3-RNA panel for AFP-negative patients, we used 8 AFP-positive (AFP > 400 ng/mL) liver cancer patients as a positive training set, as well as 26 HDs and 24 chronic hepatitis B (CHB) patients as a negative training set, to generate a random forest model. When requiring >95% specificity in the HDs of the training set (specificity: 96.2%), the model accurately predicted the identity of all AFP-positive patients (sensitivity: 100%) and discriminated liver cancer patients from most CHB patients (specificity: 91.7%) (Figure 7B). When the 18 AFP-negative patients were assessed, 14 of 18 were predicted to be positive (sensitivity: 77.8%) (Figure 7B). We also found that the specificity and sensitivity of each individual marker were inferior to those of the combination of three selected RNAs (Figure S7). To confirm this result, we assessed 30 additional AFP-negative patients as an independent validation set. The sensitivity of the validation set was 80.0% (24 of 30), which was similar to that of the testing set. Meanwhile, 77.1% (27 of 35) early stage (0 and A) cancer patients were accurately predicted in the testing and validation sets, indicating the potential of our panel in the early diagnosis of liver cancer.

## Conclusions

Based on integrative analysis of long exRNAs and multiple validation methods, this study demonstrated the potential of long exRNA variations as cancer biomarkers and revealed several candidate biomarkers for liver cancer. In particular, we revealed a 3-RNA detection panel for liver cancer patients, especially AFP-negative patients and those with early-stage cancer.

Our study shows that noncoding RNAs are able to achieve high performance whether they are assessed by RT-qPCR or analysis of sequencing data. Noncoding RNA molecules tend to form stable secondary structures that protect them from degradation, which may facilitate verification 52. However, our analysis of mRNAs did not reveal AS or differential editing events that were reliable biomarkers based on RT-qPCR. In plasma, RNAs, especially mRNAs, tend to be fragmented randomly among individuals. Thus, with different lengths, these fragments are difficult to measure accurately in different individuals with pre-manufactured sets of RT-qPCR primers.

In addition, we have shown that the three selected biomarkers in plasma do not require exosome purification prior to use as highly sensitive and specific diagnostic biomarkers for HCC, suggesting that they could be applied in a convenient and cost-effective manner. Moreover, purification steps for exosomes/MVs could introduce RNA losses and data heterogeneity among samples and batches 53, 54.

Heterogeneity between samples is an inevitable obstacle. In practical clinical applications, the levels of circulating biomarkers are affected by a variety of individual characteristics, including gender, age, ethnicity, genetic background, lifestyle, and disease history 55. Therefore, expanding the sample size to ensure the accuracy of cancer diagnosis is a crucial consideration. In addition, as with any screening procedure conducted with the goal of translating results from the bench to the bedside, the effectiveness of normalization method should be carefully considered and determined during clinical applications.

## Methods

### Cohort design for discovery and validation

The exosomal RNA-seq (exoRNA-seq) data were downloaded from exoRBase 32, including 12 colorectal cancer (CRC) patients, 21 hepatocellular carcinoma (HCC) patients, 14 pancreatic adenocarcinoma (PAAD) patients, and 32 healthy donors (HD) (Figure 3). Subsequently, we used different datasets to discover and validate the candidate biomarkers in liver cancer (Fig S1). We profiled cell free RNA-seq (cfRNA-seq) data to identify recurrently dysregulated RNA variations in the plasma of liver cancer patients compared with healthy donors (0/A stage liver cancer: 30; B/C stage liver cancer: 5; healthy: 30). In addition, RNA-seq data for 50 HCC tissues and 50 paired normal tissues in The Cancer Genome Atlas (TCGA) database were downloaded from the National Cancer Institute Cancer Genomics Hub (CGHub) 57. They were used to confirm these RNA variations at the tissue level. We first validated biomarker candidates in an internal validation cohort (0/A stage liver cancer: 13; healthy: 13), which shares the same part of samples as the cohort of cfRNA-seq. Finally, we validated these RNA variations in an independent cohort by RT-qPCR assay (0/A stage liver cancer: 50; B/C stage liver cancer: 18; healthy: 37; Chronic hepatitis B: 24). The characteristics of study participants are presented in Table S1.

### Cell free RNA (cfRNA) isolation and sequencing

Cell free RNAs (cfRNAs) were isolated from ~1 ml plasma using the Plasma/Serum Circulating RNA and Exosomal Purification kit (Norgen, cat 42800) according to the manufacturer's protocol. The DNA residuals in the cfRNAs were digested using Recombinant DNase I (RNase-free). We then cleaned up the cfRNAs using RNA Clean and Concentrator-5 kit (Zymo), and eluted them with 7 µL elution buffer.

We added 1µL External RNA Controls Consortium (ERCC) spike-in (with suitable dilution) to the 7 µL eluted cfRNAs as input to prepare the cfRNA sequencing libraries, with SMARTer Stranded Total RNAseq-Pico Input Mammalian kit (Clontech) according to the manufacturer's instructions. Finally, the RNA libraries were sequenced on Illumina XTen (2 × 150 bp) platform with depth of >10 million reads per sample.

### Exosome purification and characterization

We used 32 plasma samples (Table S1) for the exosome enrichment assay. In each experiment, plasma samples of four individuals were mixed. Then, each mixed sample was spun at 12,000g for 20 min under 4 °C and resuspended in cold PBS, filtered through a 0.22 µm filter. Cold PBS (20 mL) was added into the sample and then centrifuged (110,000g, 4 °C, 4 h). The pellet containing the exosomes was resuspended in cold PBS. We used Nanosight NS300 (Mastersizer) and transmission electron microscopy to get modal value, concentration, and size distribution of the isolated exosomes.

### RT-qPCR validation

In miRNA RT reactions and single-primer qPCR experiments, we used the miRcute Plus miRNA First-Strand cDNA Synthesis Kit and miRcute Plus miRNA qPCR Detection Kit (TIANGEN, KR211 and FP411). In long RNA RT reactions, we used TIANScript II First-Strand cDNA Synthesis Kit and FastFire qPCR PreMix (SYBR Green) Kit (TIANGEN, KR107 and FP207). Each Ct value was the average of 3 replicates, and the detection limit was lower than 0.1 pg. We list primer sequences for the RT-qPCR of long RNAs in Table S2. Particularly, the primers of the circular RNAs were designed for the junction regions. The primers for the miRNAs were the same as the published papers 58. For the validation of differentially expressed genes, primers were selected with the best performance that had single and correct amplification products. For the validation of alternatively spliced genes, three kinds of primers were designed for each gene. The PCR products of alternative splicing genes were separated by 1.5% agarose gels, stained with GelSage nucleic acid gel stain.

We used *HULC* as a positive control for lncRNAs in liver cancer 36-38; and we used 6 miRNAs as positive controls for miRNAs in liver cancer 11, 48. We used External RNA Controls Consortium (ERCC) RNA control and glyceraldehyde-3-phosphate dehydrogenase (GAPDH) as the external and internal reference for long RNAs, respectively. ERCC showed better performance in distinguishing healthy donors and HCC patients, so that we chose ERCC for further assays (Figure S6). Based on previous studies 59, we used miR-1228 rather than U6 snRNA as the internal reference for miRNAs to normalize the absolute cycle threshold (Ct) value in each sample.

### Data process on the sequencing data

We used Cutadapt to remove adapters and read pairs with an average quality score below 30 in either read of the pair. Paired reads were first mapped to the rRNA database (from NCBI RefSeq database). The unmapped reads were then mapped to the human genome sequences (UCSC genome build hg38). A genome index was generated using STAR with splicing junction annotations from GENCODE V27. Remaining reads after the first two steps were mapped to the circular RNAs from circBase. We only considered a paired reads (from 5'-end in read 1 to 3'-end in read 2) that overlapped with the back-splicing junction as circular RNA reads. Only read pairs mapped to the human genome or circular RNAs were included in further analyses. We removed duplicated read pairs using picard MarkDuplicates because the dataset contained a large proportion of duplicated reads due to PCR amplification in library construction. Finally, a gene count matrix was generated using featureCounts with options “-M -p -s 1 -t exon -g gene_id”.

### Differential expression analysis

We used DESeq2 60 to identify differentially expressed genes (DEGs) between cancer and control (i.e., HD). The significance cutoff was set at |log2(fold-change)| ≥ 1 and false discovey rate (FDR) value ≤ 0.05.

When selecting candidate events recurrently identified in both exoRNA-seq and cfRNA-seq (Figure 4A), we ranked the 122 recurrently dysregulated genes by their fold changes in cfRNA-seq in each type of RNA (mRNA, snoRNA, lncRNA, and circRNA) respectively. For mRNA, we selected the top up-regulated and the top down-regulated as candidate events. As for snoRNA, lncRNA, and circRNA, the recurrently dysregulated genes were all up-regulated in liver cancer, then we selected the top snoRNA, the top two lncRNAs, and the top three circRNAs for further experiment validation, while only two of these three circRNAs were validated by sanger sequencing.

### Differential RNA editing analysis

In order to obtain differentially edited sites, we applied RNAEditor 61 to detect RNA editing sites with default parameters. RNA editing sites with frequency larger than 20% in cancer or HD were kept for the following analyses. Wilcoxon signed rank test was applied to test the sum of the remaining RNA editing rate in each site between cancer patients and HDs. The differential RNA editing site was defined as site with an FDR value ≤ 0.05.

### Alternative splicing analysis

The alternative splicing analysis was performed by rMATS 56 with the parameter setting of: --cstat 0.0001 --libType fr-secondstrand. We applied a cutoff of FDR value ≤ 0.01 and |△PSI (average percent spliced in the index (PSI) of cancer samples - average PSI of HDs)| ≥ 0.05 to determine the altered splicing genes.

When selecting recurrent events (Figure 4C), we started from 222 alternative splicing events identified using exoRNA-seq data. However, only one event was also identified in the cfRNA-seq data, probably because the sequencing depth of cfRNA-seq was not enough for alternative splicing analysis of rMATS. Therefore, 37 out of 222 alternative splicing events were identified from cfRNA-seq data without considering FDR cut-off (21 △PSI ≥ 0.05 and 16 △PSI ≤ -0.05). We ranked the 37 events by absolute △PSI and selected three candidates whose gene function was cancer related and reported by other studies from the top 10 events.

### Alternative polyadenylation analysis

To discover alternative polyadenylation events, we used DaPars 29 with the parameter setting of: FDR_cutoff = 0.05, PDUI_cutoff = 0.2, Fold_change_cutoff = 0.59 (PDUI: percentage of distal polyA site usage index).

### Cancer gene enrichment analysis

We downloaded the cancer gene sets as positive controls from the following resources: the Network of Cancer Genes (NCG5) 62, the Atlas of Genetics and Cytogenetics in Oncology and Hematology (PosAGO) 63, the Cancer Gene Census (CGC) version 73 (PosSomatic and PosTrans) 64, UniprotKB (PosUniprotKB) 65, a query of DISEASES (PosTextMine) 66, and the PosUnionAll. PosUnionAll is the union of all these positive control lists of PosSomatic, PosTrans, PosUniprotKB, PosTextMine, and PosAGO.

We downloaded the gene sets unrelated to cancers as negative controls from the following resources: a conservative version of the negative AGO list (NegAGOClean) 67, a list derived from AGO (NegAgoFull) 63, and a list of known non-driver genes (NegDavoli) 68.

Finally, differential expression, alternative splicing, alternative polyadenylation, and differential RNA editing gene sets were overlapped with above gene sets. The ratios of the overlapped genes over the size of each gene set were calculated.

### Pathway enrichment analysis

Pathway analyses were carried out using Metascape 69. The top 20 enriched KEGG pathways based on *P-*value were annotated as tumorigenesis and metastasis, immune or metabolism-related pathways.

### Statistical analysis

All tests for comparing the expression levels in different groups were Wilcoxon rank sum tests. *P*-values of less than 0.05 were considered to be statistically significant.

### Machine learning method for combining multiple RNAs as a panel

Machine learning methods were applied to evaluate the classification performance of the selected features. We used cross-validation for model selection. Samples were subjected to 5-fold cross-validation with 10 repeats (RepeatedStratifiedKFold in the scikit-learn package), yielding 50 class-balanced independent training and testing sets in an 80% - 20% manner. AUROC (Area Under the Receiver Operating Characteristic curve) was utilized as the performance metric. We applied a grid search strategy (GridSearchCV in scikit-learn package) to select hyper-parameters that maximize the classification score (score method of classifiers in scikit-learn package) (Supplementary Figure 5). The implementation of each classifier was listed below: Decision Tree (sklearn.tree.DecisionTreeClassifier); Logistic Regression (sklearn.linear_model.LogisticRegression); SVM (sklearn.svm.SVC); Random Forest (sklearn.ensemble.RandomForestClassifier). We used Random Forest as the default classifier.

To compare and select an optimal feature set among 11 features (5 ncRNAs and 6 miRNAs) in the RT-qPCR data sets, we traversed feature numbers of 3, 5, 7, 9, and all of the corresponding feature combinations (1,201 in total). The combination with the highest average AUC among all possible combinations was selected. Due to the instability of random partition and small sample size, we averaged the results of 50 cross-validations to obtain a ROC curve and measured the prediction performance of different feature combinations.

Finally, a Random Forest model-based panel with the selected 3 features (a circRNA, a snoRNA, and a miRNA) was trained on the AFP-positive samples, tested and validated on the AFP-negative samples. The original RT-qPCR values were scaled to 0-1 in the machine learning model-based panel.

## Supplementary Material

Supplementary figures and tables.

## Abbreviations

exRNA: extracellular RNA
exoRNA-seq: exosomal RNA-seq
cfRNA-seq: cell-free RNA-seq
cfRNA: cell free RNA
MV: microvesicle
RNP: ribonucleic acid protein
NSCLC: non-small cell lung cancer
HBV: hepatitis B virus
HCC: hepatocellular carcinoma
CRC: colorectal cancer
PAAD: pancreatic adenocarcinoma
HD: healthy donor
snRNA: small nuclear RNA
snoRNA: small nucleolar RNA
tRNA: transfer RNA
circRNA: circular RNA
mRNA: messenger RNA
lncRNA: long noncoding RNA
AS: alternative splicing
APA: alternative polyadenylation
AFP: alpha feto-protein
FDR: false discovery rate
EV: extracellular vesicle
AUROC: Area Under the Receiver Operating Characteristic curve
CHB: chronic hepatitis B
TCGA: The Cancer Genome Atlas
CGHub: Cancer Genomics Hub
ERCC: External RNA Controls Consortium
GAPDH: glyceraldehyde-3-phosphate dehydrogenase
Ct: cycle threshold
DEG: differentially expressed gene
FDR: false discovery rate
PSI: percent spliced in index
PDUI: percentage of distal polyA site usage index
