# Supplementary material for: Integrative analysis of long extracellular RNAs reveals a detection panel of noncoding RNAs for liver cancer
Source: Theranostics. 2021 Jan 1;11(1):181–93. doi: 10.7150/thno.48206 (PMC7681086; doi:10.7150/thno.48206)
Supplement: Supplementary file 1 — Supplementary figures and tables. [file thnov11p0181s1.pdf]

# Supplementary Information

**Integrative analysis of long extracellular RNAs reveals a detection panel of structured and circular RNAs for liver cancer**


**Supplementary Tables** 2

**Table S1 | Clinical characteristic of study participants** 2

**Table S2 | Gene primer sequences used in RT-qPCR for long RNAs** 3

**Supplementary Figures** 4

**Figure S1 | Overview of integrative analysis and experimental design in liver cancer.** 4

**Figure S2 | Characteristics of cell-free RNA-seq and exosomal RNA-seq.** 5

**Figure S3 | Alternative polyadenylation and differential editing events in liver cancer.** 6

**Figure S4 | RT-qPCR validation of differential expression and alternative splicing RNAs in the same 26 samples as used in the cfRNA-seq dataset.** 7

**Figure S5 | Machine learning model performances on the 10 candidates in exoRNA-seq and cfRNA data** 8

**Figure S6 | Comparison of using External RNA Controls Consortium (ERCC) RNA control and glyceraldehyde-3-phosphate dehydrogenase (GAPDH) as reference genes for RT-qPCR analysis.** 9

**Figure S7 |** ***miR-122*** **expression level for the diagnosis of AFP-negative liver cancer** 10

# Supplementary Tables

**Table S1 | Clinical characteristic of study participants**

Clinical characteristic of participants in this study involved in the cfRNA sequencing, RT-qPCR, and exosome enrichment assay, including age, sex, stage, tumor size, vascular invasion, AFP level, and category/tumor site.

## Table1. Clinical characteristic of study participants.

| *cf-RNA Sequencing* | | | | | |
|---|---|---|---|---|---|
| **Healthy Donor (n = 30)** | | | **Liver Cancer (n = 35)** | | |
| **Variable** | **No.** | **%** | **Variable** | **No.** | **%** |
| Age, years | | | Age, years | | |
| Mean | 66 | | Mean | 56 | |
| SD | 7 | | SD | 12 | |
| Sex | | | Sex | | |
| Male | 14 | 47% | Male | 29 | 83% |
| Female | 16 | 53% | Female | 6 | 17% |
| | | | Stage | | |
| | | | 0 | 4 | 11% |
| | | | A | 26 | 75% |
| | | | B | 4 | 11% |
| | | | C | 1 | 3% |
| | | | Tumor size | | |
| | | | ≤ 3 cm | 11 | 31% |
| | | | > 3 cm | 24 | 69% |
| | | | Vascular Invasion | | |
| | | | Yes | 2 | 6% |
| | | | No | 33 | 94% |
| | | | No biopsy | 0 | 0 |
| | | | AFP | | |
| | | | ≤400ng/ml | 28 | 80% |
| | | | > 400ng/ml | 7 | 20% |
| | | | Category/Tumor Site | | |
| | | | HCC | 33 | 94% |
| | | | ICC | 0 | 0 |
| | | | Others | 2 | 6% |

| *RT-qPCR* | | | | | |
|---|---|---|---|---|---|
| **Healthy Donor (n = 37)** | | | **Liver Cancer (n = 68)** | | |
| **Variable** | **No.** | **%** | **Variable** | **No.** | **%** |
| Age, years | | | Age, years | | |
| Mean | 62 | | Mean | 56 | |
| SD | 13 | | SD | 9 | |
| Sex | | | Sex | | |
| Male | 15 | 41% | Male | 61 | 90% |
| Female | 22 | 59% | Female | 7 | 10% |
| | | | Stage | | |
| | | | 0 | 0 | 0 |
| **Chronic hepatitis B * (n = 24)** | | | A | 57 | 84% |
| | | | B | 6 | 9% |
| **Variable** | **No.** | **%** | C | 5 | 7% |
| Age, years | | | Tumor size | | |
| Mean | 52 | | ≤ 3 cm | 28 | 41% |
| SD | 11 | | > 3 cm | 40 | 59% |
| Sex | | | Vascular Invasion | | |
| Male | 11 | 46% | Yes | 3 | 5% |
| Female | 13 | 54% | No | 64 | 94% |
| | | | No biopsy | 1 | 1% |
| | | | AFP | | |
| | | | ≤400ng/ml | 58 | 85% |
| | | | > 400ng/ml | 10 | 15% |
| | | | Category/Tumor Site | | |
| | | | HCC | 67 | 99% |
| | | | ICC | 0 | 0 |
| | | | Others | 1 | 1% |

| *Exosome Enrichment Assay* | | | | | |
|---|---|---|---|---|---|
| **Healthy Donor (n = 16)** | | | **Liver Cancer (n = 16)** | | |
| **Variable** | **No.** | **%** | **Variable** | **No.** | **%** |
| Age, years | | | Age, years | | |
| Mean | 62 | | Mean | 57 | |
| SD | 9 | | SD | 10 | |
| Sex | | | Sex | | |
| Male | 8 | 50% | Male | 13 | 81% |
| Female | 8 | 50% | Female | 3 | 19% |
| | | | Stage | | |
| | | | 0 | 0 | 0 |
| | | | A | 9 | 56% |
| | | | B | 7 | 44% |
| | | | C | 0 | 0 |
| | | | Tumor size | | |
| | | | ≤ 3 cm | 4 | 25% |
| | | | > 3 cm | 12 | 75% |
| | | | Vascular Invasion | | |
| | | | Yes | 1 | 6% |
| | | | No | 15 | 94% |
| | | | No biopsy | 0 | 0 |
| | | | AFP | | |
| | | | ≤400ng/ml | 11 | 80% |
| | | | > 400ng/ml | 5 | 20% |
| | | | Category/Tumor Site | | |
| | | | HCC | 15 | 94% |
| | | | ICC | 1 | 6% |
| | | | Others | 0 | 0 |

*: CHB patients were only used in the analysis of Figure 7 and Supplementary Figure 7.

**Table S2 | Gene primer sequences used in RT-qPCR for long RNAs**

| Gene | Forward Primer | Reverse Primer |
|---|---|---|
| *UGT2B7* | CAGCAACTGGAAAACAAGCA | CTTTCCACAATTCCCAGAGC |
| *CAMK4* | GATGGCAACGAGGACATGAAA | AACTTTCTCTAAGGCTTGCACC |
| *circ-0073052* | TCACCAGCCAACAGCTAAGA | TTTGTAACTGTCACACTTCTCC |
| *circ-0080695* | TGTGCGCCATAAGGAATTTCAA | ACTCCTCCTGGGTGAAATCCA |
| *HULC* | ACTCTGAAGTAAAGGCCGGA | TGCCAGGAAACTTCTTGCTTG |
| *LINC01226* | CCAGAGCTTCACACAAGCTATC | GAAGCCCCCTCCAATGTC |
| *SNORD3B-1* | GAGAAGTTTCTCTGAACGTGTA | AATGGCTGACGGCAGTTG |
| *ADD3* (Full length) | GAACGTAAACAACAAGGCCT | TACCATGACAGGCACTTCCA |
| *ADD3* (Non-ES) | GCCTAGAAGGATGCTGAGCA | CAGCTCATGGTTTTCCTTCTAAT |
| *ADD3* (ES) | AGGCCTAGAAGGAAAACCATGA | ATCCTTGCCATTTACTACCATG |
| *HNRNPH1* (Full length) | GGAAACCTACATCGTTCCTTCT | CAAGATTGAAGTCGAGATGACG |
| *HNRNPH1* (Non-ES) | AAGAAGAGTCCCCCTCCTCATG | AAAGTTATATGGGACACGGGAT |
| *HNRNPH1* (ES) | GAAACATGCCGAATCTCCT | AAAGTTATATGGGACTCTTCTTG |
| *UBE2B1* (Full length) | GACCGCTAGTGAGTATATCGTG | GTCGATTGACACGGTAGAGGA |
| *UBE2B1* (Non-ES) | GACATAGTGACACGTATCCTCG | CACCGACGCCGTACTCCT |
| *UBE2B1* (ES) | AGGAGTACGGCTCATTTTCA | TTGACACGGTAGAGGATGAGGA |
| *GAPDH* | GAACGGGAAGCTTGTCATCAA | ATCGCCCCACTTGATTTTGG |
| *ERCC96* | CAACGGTGCAATCTCAGCTA | CACGAGGATGTTCCTGTTGA |

# Supplementary Figures

**Figure S1 | Overview of integrative analysis and experimental design in liver cancer.**
We used 3 discovery sets (exoRNA-seq data from exoRBase, tissue RNA-seq data from TCGA, and self-profiled cfRNA-seq data) to discover candidate biomarkers, and a validation set (RT-qPCR data) to validate the marker in an independent cohort. Multiple types of RNA regulatory events were assayed on exosomal RNA-seq (exoRNA-seq) data to identify RNA variations. Cell-free RNA-seq (cfRNA-seq) data were profiled to find recurrent RNA variations as candidate biomarkers. TCGA data were collected to confirm these candidate biomarkers at tissue level. RT-qPCR experiment validated these candidate biomarkers in an independent cohort. The patients of liver cancer are in four stages: 0, A, B and C.

(HD: Healthy Donors, HCC: hepatocellular carcinoma, CHB: Chronic hepatitis B, *: CHB patients' data were only used in the analysis of Figure 7 and Supplementary Figure 7.)

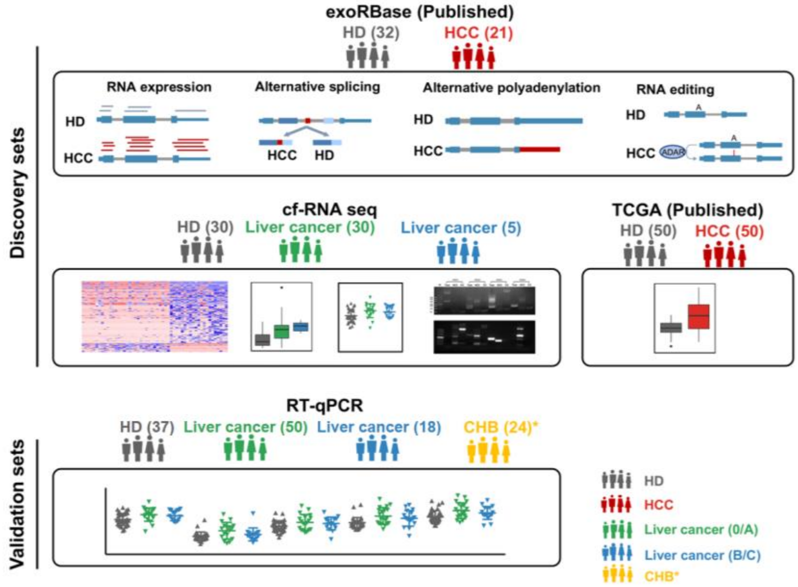

**Figure S2 | Characteristics of cell-free RNA-seq and exosomal RNA-seq.**

(A) Left panel, number of genes identified by cell-free RNA-seq belonging to each RNA species. Right panel, reads distribution of RNA species among all individuals by cell-free RNA-seq.

(B) Left panel, number of genes identified by exosomal RNA-seq belonging to each RNA species. Right panel, reads distribution of RNA species among all individuals by exosomal RNA-seq.

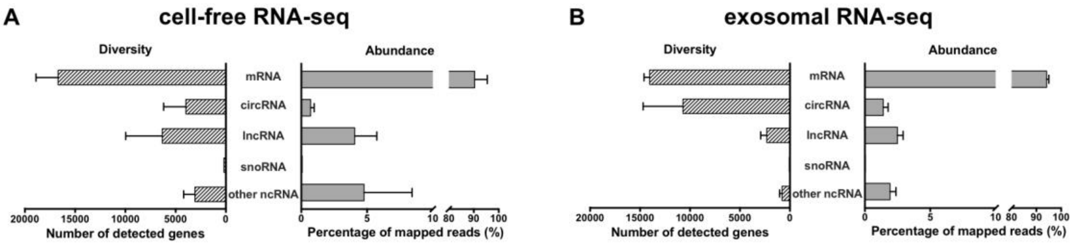

**Figure S3 | Alternative polyadenylation and differential editing events in liver cancer.**

(A) Alternative polyadenylation events between HCC patients and healthy donors identified in exoRNA-seq data.

(B) Differential editing events between HCC patients and healthy donors identified in exoRNA-seq.

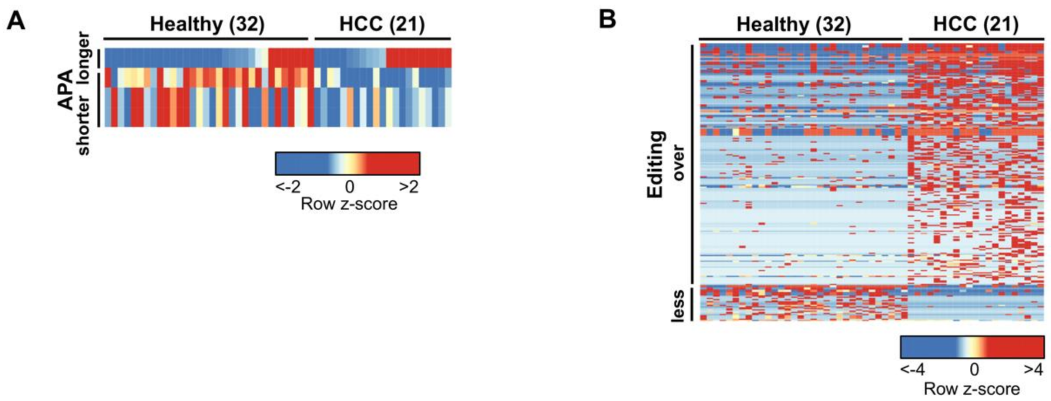

**Figure S4 | RT-qPCR validation of differential expression and alternative splicing RNAs in the same 26 samples as used in the cfRNA-seq dataset.**

(A) Validation of the seven selected differentially expressed RNA candidates by sequencing data and RT-qPCR results. The RT-qPCR samples are a part of the samples of sequencing data (13 0/A stage liver cancer patients; 13 healthy donors). ***: *P*-value < 0.001, **: *P*-value < 0.01, *: *P*-value < 0.05, Wilcoxon rank sum test. (FC: fold-change)

(B) Validation of the three selected alternatively spliced RNA candidates by sequencing data and RT-qPCR results. The RT-qPCR samples are a part of the samples of sequencing data (13 0/A stage liver cancer patients; 13 healthy donors). ***: *P*-value < 0.001, **: *P*-value < 0.01, *: *P*-value < 0.05, Wilcoxon rank sum test. (PSI: percent spliced in index)

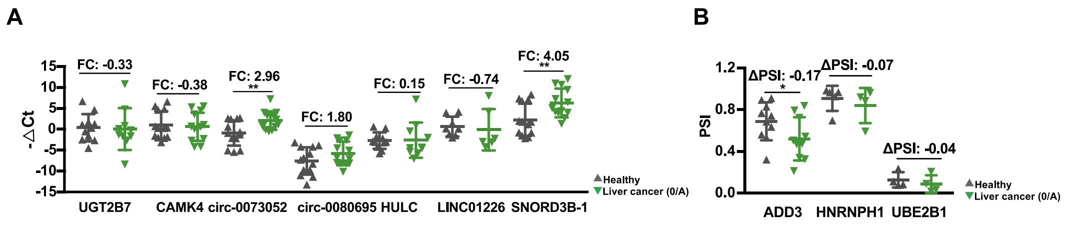

**Figure S5 | Machine learning model performances on the 10 candidates in exoRNA-seq and cfRNA data**

(A) The performance of different classifiers (DT: Decision Tree, SVM: Support Vector Machine, LR: Logistic Regression, RF: Random Forest) on 10 candidates (7 differentially expressed RNAs and 3 alternative splicing events) in exoRNA-seq and cfRNA-seq data. We used 5-fold cross-validation and repeated 10 times by re-shuffling the data.

(B) The left ROC curve represents the RF model trained on cfRNA-seq data and tested on exoRNA-seq data. AUC values of all stages (0, A, B and C) is labeled for exoRNA-seq. (The exoRNA-seq data do not have stage information.) The right ROC curve represents the RF model trained on exoRNA-seq data and tested on cfRNA-seq data. AUC values of all stages (0, A, B and C) and early stages (0 and A) are labeled for cfRNA-seq.

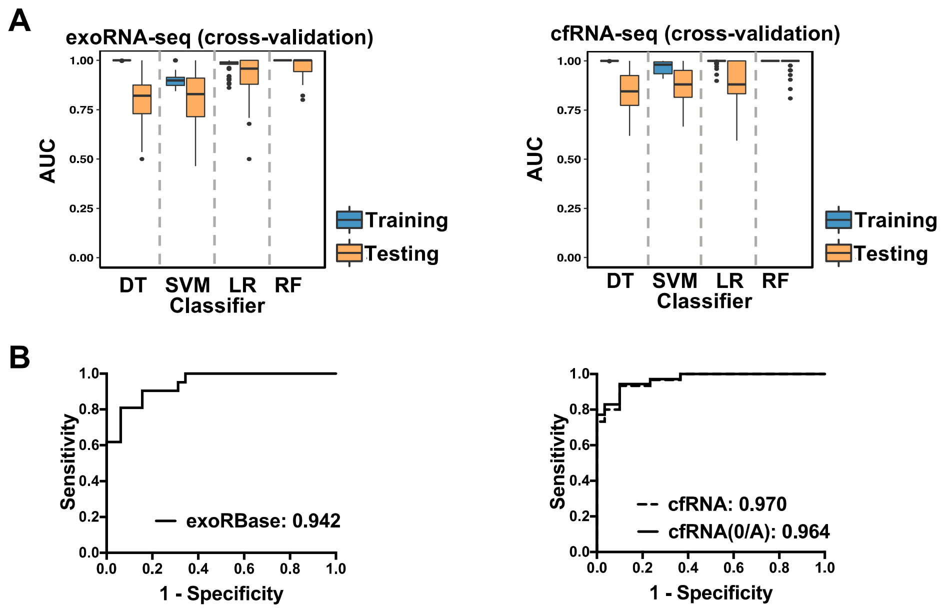

**Figure S6 | Comparison of using External RNA Controls Consortium (ERCC) RNA control and glyceraldehyde-3-phosphate dehydrogenase (GAPDH) as reference genes for RT-qPCR analysis.**

(A) Fold change values of expression level by RT-qPCR experiment for the selected five genes using ERCC and GAPDH as indicators between liver cancer patients (0/A stage) and healthy donors, liver cancer patients (B/C stage) and healthy donors.

(B) P-values of Wilcoxon rank sum test of the expression level by RT-qPCR experiment for the selected five genes using ERCC and GAPDH as indicators between liver cancer patients (0/A stage) and healthy donors, liver cancer patients (B/C stage) and healthy donors.

**A**

| | | circ-73052 | circ-80695 | HULC | LINC01226 | SNORD3B-1 |
|---|---|---|---|---|---|---|
| ERCC | Liver cancer (0/A) | 2.137 | 2.508 | 1.970 | 2.893 | 2.815 |
| | Liver cancer (B/C) | 1.639 | 0.842 | 1.478 | 1.968 | 1.771 |
| GAPDH | Liver cancer (0/A) | -0.269 | -0.670 | -0.038 | 0.734 | 0.186 |
| | Liver cancer (B/C) | 0.877 | 0.073 | 1.569 | 1.582 | 1.327 |

Fold Change: -1, 0, 3

**B**

| | | circ-73052 | circ-80695 | HULC | LINC01226 | SNORD3B-1 |
|---|---|---|---|---|---|---|
| ERCC | Liver cancer (0/A) | 0.005 | 0.010 | 0.031 | 0.002 | 0.002 |
| | Liver cancer (B/C) | 0.022 | 0.068 | 0.020 | 0.002 | 0.010 |
| GAPDH | Liver cancer (0/A) | 0.523 | 0.788 | 0.581 | 0.084 | 0.326 |
| | Liver cancer (B/C) | 0.268 | 1.08E-08 | 0.014 | 1.30E-04 | 0.003 |

P-value: 1, 0.05, 0

**Figure S7 | *miR-122* expression level for the diagnosis of AFP-negative liver cancer**

We use *miR-122* as an example to show that individual marker is not as good as the 3-RNA panel. Although *miR-122* was reported as biomarker for hepatic diseases (e.g., CHB: Chronic hepatitis B), the researches on the detection in chronic viral hepatitis have been inconsistent.[1] Thus, a single marker alone is not as good as the combination of 3 RNAs.

-△Ct values of the *miR-122* expression level by RT-qPCR experiment panel in all samples. The cutoff of -△Ct value is y =0.96, defined by requiring > 95% specificity in the training set (healthy donors, chronic hepatitis B patients and liver cancer patients). (AFP+: AFP positive (AFP > 400 ng/ml) liver cancer patients, AFP-: AFP negative (AFP < 400 ng/ml) liver cancer patients)

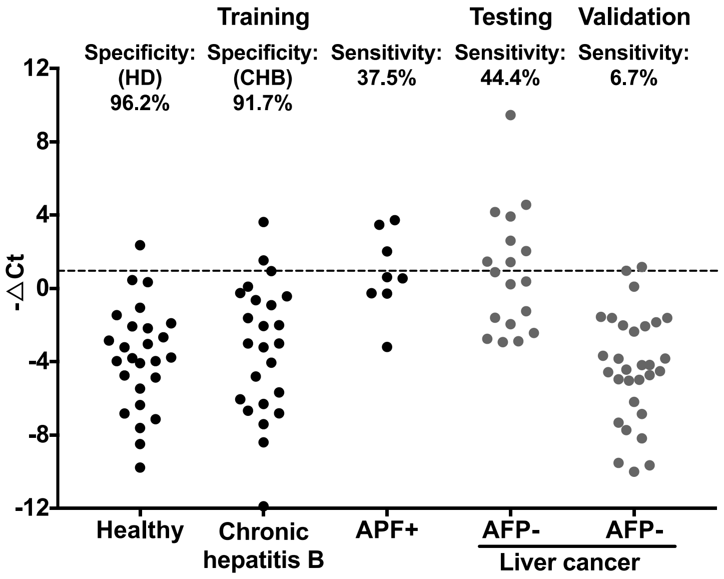


Reference

1 Zhou, X. *et al.* Diagnostic value of circulating miRNA-122 for hepatitis B virus and/or hepatitis C virus-associated chronic viral hepatitis. *Biosci Rep* **39**, doi:10.1042/BSR20190900 (2019).
